# Determining immune components necessary for progression of pigment dispersing disease to glaucoma in DBA/2J mice

**DOI:** 10.1186/1471-2156-15-42

**Published:** 2014-03-28

**Authors:** K Saidas Nair, Jessica Barbay, Richard S Smith, Sharmila Masli, Simon WM John

**Affiliations:** 1 Howard Hughes Medical Institute, The Jackson Laboratory, Bar Harbor, ME, USA; 2 Department of Ophthalmology, University of California, San Francisco, CA, USA; 3 Department of Ophthalmology, Boston University, Boston, MA, USA; 4 Department of Ophthalmology, Tufts University of Medicine, Boston, MA, USA

**Keywords:** Glaucoma, Pigmentary glaucoma, Mouse model, DBA/2J, CD94, NK cells, ACAID, Iris disease, Intraocular pressure

## Abstract

**Background:**

The molecular mechanisms causing pigment dispersion syndrome (PDS) and the pathway(s) by which it progresses to pigmentary glaucoma are not known. Mutations in two melanosomal protein genes (*Tyrp1*^*b*^ and *Gpnmb*^*R150X*^) are responsible for pigment dispersing iris disease, which progresses to intraocular pressure (IOP) elevation and subsequent glaucoma in DBA/2J mice. Melanosomal defects along with ocular immune abnormalities play a role in the propagation of pigment dispersion and progression to IOP elevation. Here, we tested the role of specific immune components in the progression of the iris disease and high IOP.

**Results:**

We tested the role of NK cells in disease etiology by genetically modifying the B6.D2-*Gpnmb*^*R150X*^*Tyrp1*^*b*^ strain, which develops the same iris disease as DBA/2J mice. Our findings demonstrate that neither diminishing NK mediated cytotoxic activity (*Prf1* mutation) nor NK cell depletion (*Il2rg* mutation) has any influence on the severity or timing of *Gpnmb*^*R150X*^*Tyrp1*^*b*^ mediated iris disease. Since DBA/2J mice are deficient in CD94, an important immune modulator that often acts as an immune suppressor, we generated DBA/2J mice sufficient in CD94. Sufficiency of CD94 failed to alter either the iris disease or the subsequent IOP elevation. Additionally CD94 status had no detected effect on glaucomatous optic nerve damage.

**Conclusion:**

Our previous data implicate immune components in the manifestation of pigment dispersion and/or IOP elevation in DBA/2J mice. The current study eliminates important immune components, specifically NK cells and CD94 deficiency, as critical in the progression of iris disease and glaucoma. This narrows the field of possible immune components responsible for disease progression.

## Background

Glaucoma is a group of diseases in which retinal ganglion cell death and optic nerve degeneration lead to blindness. High intraocular pressure (IOP) is a major risk factor contributing to glaucoma [1]. Pigment dispersion syndrome (PDS) is a common condition that results in the dispersion of iris pigment into the anterior chamber (AC) [2,3]. The dispersed pigment accumulates within the ocular drainage structures, resulting in IOP elevation and glaucoma in some but not all individuals with PDS [4-8]. The molecular mechanisms causing PDS and the pathway(s) by which it progresses to pigmentary glaucoma are not known.

DBA/2J (D2) mice provide a model of inherited glaucoma. They develop a pigmentary form of glaucoma characterized by a pigment-dispersing iris disease, increased IOP, and optic nerve degeneration [9-12]. Mutations in two D2 genes [tyrosinase-related protein 1, *Tyrp1*, and glycoprotein (transmembrane) nmb, *Gpnmb*] induce the depigmenting iris disease [13,14]. Although more severe than in most human patients, components of the D2 phenotype have strong similarities to human PDS. The *Gpnmb* mutation induces prominent pigment dispersion (PD) with a radial slit-like pattern of transillumination defects and atrophy of the iris pigment epithelium, all of which are hallmarks of human PDS [14].

The *Gpnmb* gene encodes a heavily glycosylated protein that is present in multiple cell types with lysosomal-related organelles, including iris cells, dendritic cells, macrophages, NK cells and T cells [15-17]. The *Tyrp1* gene encodes a melanosomal protein with both enzymatic and structural functions [18-20]. Normally, melanosomes sequester cytotoxic intermediates produced during melanin production. GPNMB and TYRP1 are both transmembrane melanosomal proteins and their mutations result in leakage of the cytotoxic intermediates from melanosomes, thus leading to degeneration of iris cells and dispersion of pigment [14].

In addition to the iris, the presence of a mutant allele of *Gpnmb* in bone-marrow derived cell lineages is necessary for propagation of the iris disease and the subsequent elevation of IOP in D2 eyes [21,22]. Since most immune cells are bone marrow derived, these data suggested a role of immune cell dysfunction in D2 glaucoma. Although D2 eyes lack clinical signs of obvious inflammation (redeye and flare), they do exhibit a chronic and mild form of inflammation characterized by loss of ocular immune privilege. Both innate and adaptive immune privilege are compromised in D2 eyes [21], and immune processes drive the propagation of the iris disease and overall level of iris depigmentation [21]. Given that melanosomal proteins are themselves immunogenic [23], it is possible that dispersed pigment may initiate immune activation to directly inflict an inflammatory attack on the iris. Thus, melanosomal defects and the immune system may synergize to cause severe iris depigmentation and atrophy. Deficiency of T and B cells (adaptive immune cells) had no affect on *Tyrp1*^*b*^*Gpnmb*^*R150X*^ driven iris disease, suggesting that components of innate immunity contribute to the iris disease [22]. NK cells are an integral component of innate immunity. NK cells are implicated in loss of self-tolerance and can induce disease by directly inflicting a cytotoxic response against self-tissues. By virtue of their role in supporting inflammatory responses, NK cells have been postulated to participate in the pathogenesis of human diseases such as arthritis and multiple sclerosis [24].

DBA/2J mice are deficient in CD94, a molecule primarily expressed in NK cells and a small subset of CD8 T cells [25]. CD94 can function both as a suppressor or activator of immune responses. A recent study suggests that lack of an immunosupressive function mediated by CD94 may contribute to the disrupted ocular immune privilege and progression of D2 glaucoma [26]. CD94 is present on the cell surface as a homodimer or as a heterodimer associated with various NKG2 natural killer cell receptor family isoforms. The differential activity of CD94 as an immune activator or as an immune suppressor is dependent on which isoform of NKG2 serves as its interacting partner [27]. The ligation of CD94/NKG2A receptors aids in suppression of inflammatory responses [27,28]. The ligand for CD94/NKG2 is HLA-E in human and its homolog Qa1 in mouse, which are both nonclassical class I molecules [29,30]. The anti-inflammatory function of CD94 together with its deficiency in D2 mice makes it an attractive candidate to modulate the pathogenesis of the depigmenting iris disease and/or the subsequent glaucoma.

Here, we have tested if *Tyrp1*^*b*^*Gpnmb*^*R150X*^ driven iris disease is dependent on NK cell functions. Additionally, we tested if CD94 deficiency participates in the pathogenesis of distinct stages of D2 glaucoma. Identification of the molecular mechanisms causing pigment dispersion and the pathway(s) by which it progresses to pigmentary glaucoma is crucial for pinpointing processes that can be targeted by treatments to prevent glaucoma progression.

## Results

### Debilitating NK cell function does not alter iris disease

**Figure 1:**
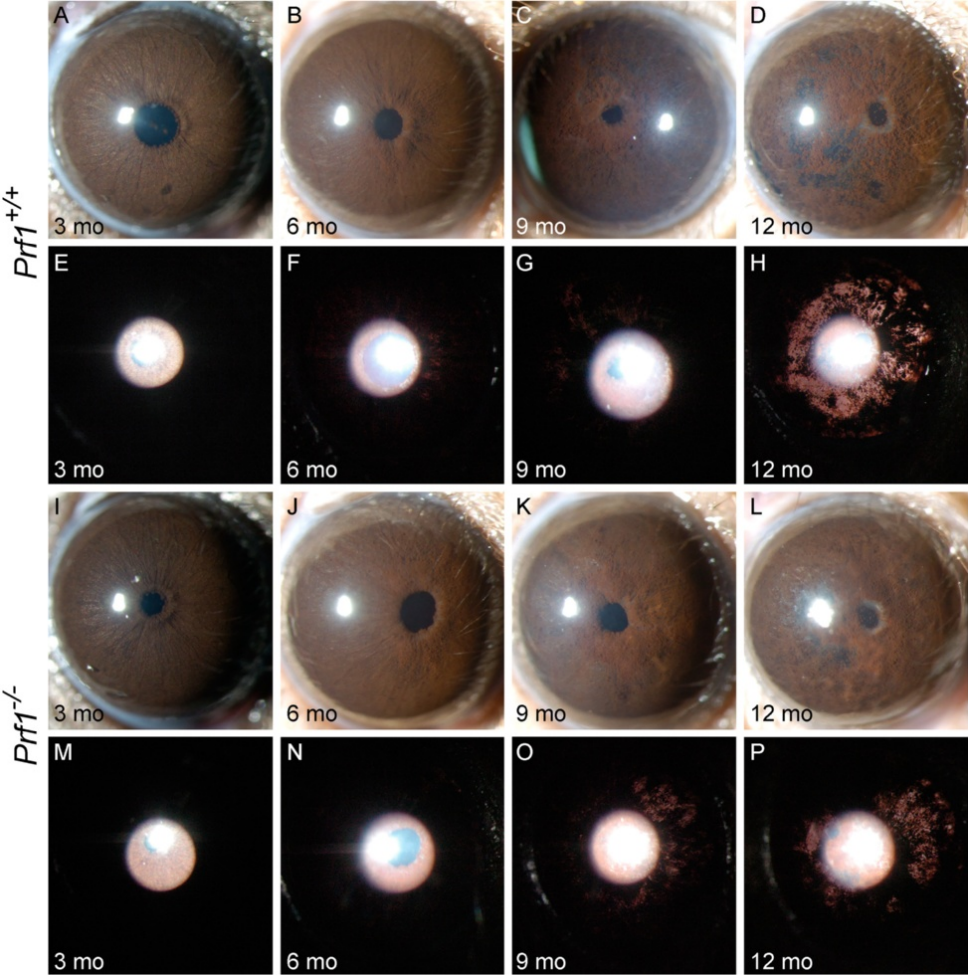
**Loss of NK cell activity has no influence on DBA/2J iris disease.** Despite some variability from eye to eye, the range and prevalence of phenotypes at each age was indistinguishable between genotypes. The top two panels are representative eyes of B6.D2-*Tyrp1*^*b*^*Gpnmb*^*R150X*^*Prf1*^*+/+*^ mice **(A-H)** at the indicated ages, and the bottom two panels of eyes of B6.D2-*Tyrp1*^*b*^*Gpnmb*^*R150X*^*Prf1*^-/-^ mice **(I-P)**. The top rows of each panel shows broad beam illumination to assess the presence of dispersed pigment within the anterior chamber and iris stromal morphology **(A-D and I-L)**. The bottom rows of each panel shows transillumination **(E-H and M-P)**. This assays the degree of iris depigmentation, detectable as areas within the image where reflected light passes through the iris. The onset and progression of iris disease in the perforin mutants are similar to that in wild-type mice: eyes of young perforin mutant mice have healthy irides **(I,M)**. At 6 mo, mutant mice exhibit mild iris disease characterized by swelling of the peripupillary region **(J)**. At 9 mo, the peripupillary region becomes atrophic, transillumination is prominent **(K,O)**. At 12 mo, there is an increasing degree of iris atrophy including distinct iris holes, profound transillumination and pigment dispersion **(L,P)**.

**Figure 2:**
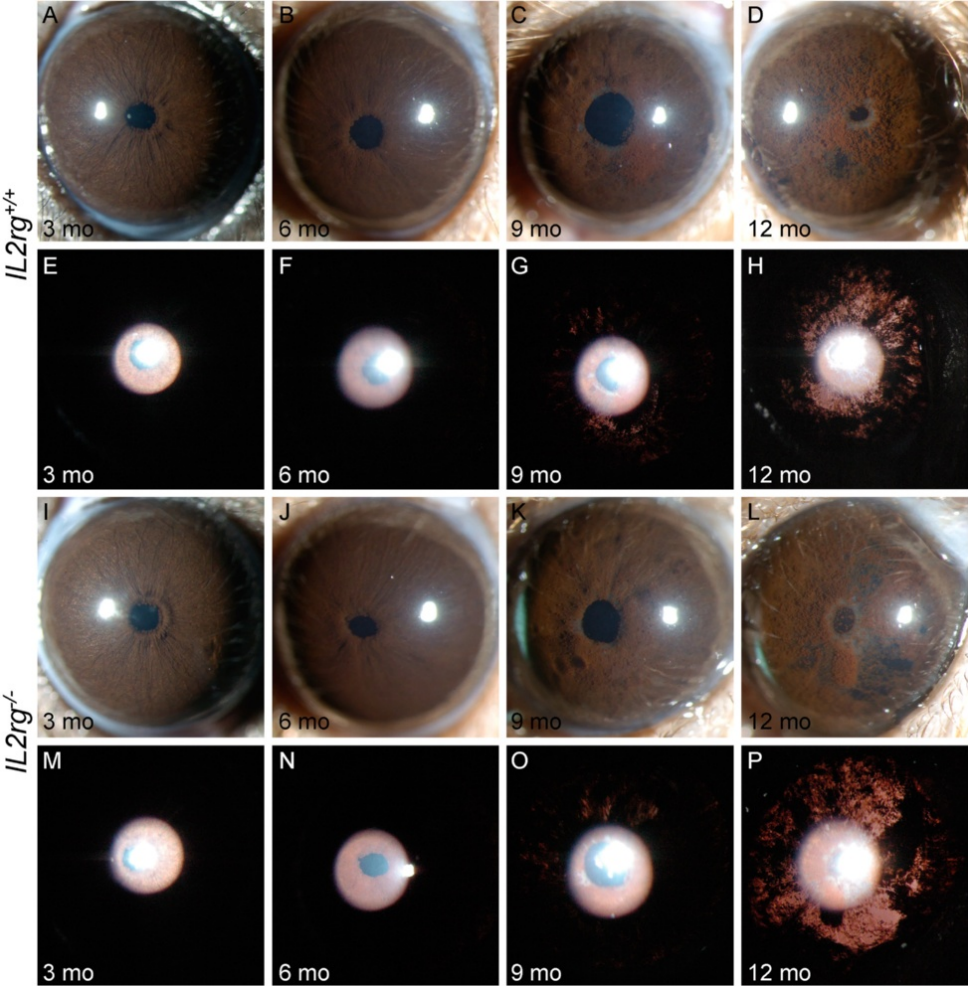
**Reduced NK cell number has no influence on *Tyrp1***^***b***^***Gpnmb***^***R150X***^**mediated iris disease.** Despite some variability from eye to eye, the range and prevalence of phenotypes at each age was indistinguishable between genotypes. The first row for the indicated genotype shows broad beam illumination to assess the dispersed pigment phenotype and the iris stromal morphology. The second row shows transillumination patterns assaying for the degree of iris depigmentation. The onset and progression of iris disease in B6.D2-*Gpnmb*^*R150X*^*Tyrp1*^*b*^*Il2rg*^*-/-*^ (I to P) is similar to that in B6.D2-*Gpnmb*^*R150X*^*Tyrp1*^*b*^*Il2rg*^*+/+*^ mice **(A-H)**. Eyes of young B6.D2-*Gpnmb*^*R150X*^*Tyrp1*^*b*^*Il2rg*^*-/-*^ mice exhibit healthy irides **(I,M)**. At 6 mo, mutant mice exhibit characteristic swelling of the peripupillary region **(J)**. At 9 mo the iris becomes atrophic, a transillumination defect is visible, and prominent dispersed pigment is visualized **(K,O)**. At 12 mo, the degree of iris atrophy is prominent as visualized by presence of distinct iris holes and severe transillumination and pigment dispersion **(L,P)**.

Existing data strongly implicates innate immunity in the progression of iris disease and subsequent IOP elevation that occurs in D2 eyes. Because NK cells are an important component of the innate immune system, we assessed if altered NK cell activity participates in the propagation of *Tyrp1*^*b*^*Gpnmb*^*R150X*^ driven iris disease. To test the role of NK cells in promoting pigment dispersing iris disease, we introduced mutations that diminish NK cell function into the B6.D2-*Gpnmb*^*R150X*^*Tyrp1*^*b*^ strain. The B6.D2-*Gpnmb*^*R150X*^*Tyrp1*^*b*^ strain consists of C57BL/6J mice that are congenic for the D2 *Gpnmb*^*R150X*^ and *Tyrp1*^*b*^ mutations. This strain develops a pigment dispersing iris disease with near identical features to that of D2 mice but is resistant to IOP elevation [31]. Since many immune mutations are available on the C57BL/6J strain background, this strain is valuable for determining the roles of specific immune processes in the pigment dispersing iris disease. CD94 is intact in this strain. Thus, it allows any effects of introduced mutations on NK cells and the iris disease to be tested without any confounding influence of insufficiency of CD94. We have introduced two mutations that influence NK cells into the B6.D2-*Gpnmb*^*R150X*^*Tyrp1*^*b*^ strain. We have tested the effects of being homozygous null for perforin (*Prf1*^*-/-*^) or homozygous null for IL2 receptor gamma (*Il2rg*^-/-^). *Prf1*^*-/-*^ mice have suppressed NK cell mediated cytotoxic function [32], and *Il2rg*^-/-^ mice are deficient in NK cells [33]. The *Il2rg*^-/-^ mice also lack mature T and B cells [33]. We have previously shown that that lack T and B cells does not alter the iris disease by using a *Rag1* mutation [22]. Therefore, any observed alteration of iris disease in *Il2rg*^-/-^ mice would most likely reflect NK cell involvement. Neither the *Prf1* or *Il2rg* mutations had any influence on the iris disease, which was indistinguishable from that of their wild-type littermates (Figures 1 and 2). Thus, neither reduced NK cytotoxic activity nor depletion of NK cells alters the onset or severity of iris disease, suggesting that NK cells do not mediate this phenotype.

### CD94 sufficiency fails to rescue the ability of antigen presenting cells (APC) to support ACAID

The CD94/NKG2A receptor is critical for induction and immunosupression by CD8 regulatory T cells (Treg) in anterior chamber associated immune deviation (ACAID) [26]. A key component of ocular immune privilege, ACAID, is a form of immune tolerance guided by multiple afferent and efferent cells [34]. This immune deviation is initiated by F4/80+ ocular APCs that capture antigens in the eye and migrate via the blood to the spleen. At that site, these APCs generate a population of Tregs capable of inhibiting Th1-mediated inflammatory immune responses such as a delayed type hyper-reactivity (DTH). It has been suggested that antigen presentation by Qa-1^b^, a non-classical MHC I molecule on the surface of APCs and its interaction with CD94/NKG2A receptor on CD8^+^ Treg T cells is important in the induction of ACAID [26]. The deficiency of CD94 disrupts CD8 Treg induction by Qa-1^b^ expressing APCs from the D2 eyes with a consequential loss of ACAID.

Additionally, we previously reported that an inherent defect in APCs from D2 mice leads to an inability to support ACAID, which may contribute to disrupted ocular immune privilege and the progression of glaucoma [22]. Subsequently, it was demonstrated that D2 mice receiving intracameral antigen have a reduced frequency of Qa-1^b^ (CD94 ligand) expressing cells in the iris and among the F4/80+ APCs in the spleen compared to a haplotype matched DBA/2NCr strain that is sufficient in CD94 [26]. Thus, a decrease in Qa-1^b^ restricted antigen presentation by APCs is likely to impede generation of immunosuppressive CD8 Tregs and may underlie failure of D2 APCs to support ACAID. We hypothesize that CD94 plays a key role in the maintenance of ocular immune privilege and its absence contributes to progression of glaucoma in D2 eyes.

**Figure 3:**
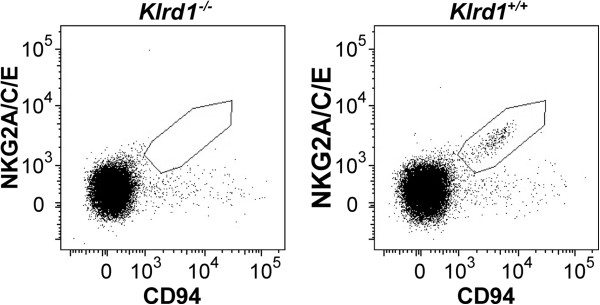
**Flowcytometric assessment of CD94 expression.** Comparison of CD94/NKG2 expressing cells on peripheral blood mononuclear cell (PBMC) from D2 *Klrd1*^*+/+*^and D2 *Klrd1*^*-/-*^ mice (the *Klrd1* gene encodes CD94). A subset of the lymphocytes from D2 *Klrd1*^*+/+*^ mice express the CD94/NKG2 receptor complex (within the drawn area). In contrast cells from D2 *Klrd1*^*-/-*^ did not express the receptor complex. The data is representative of three independent experiments.

To test the role of the CD94/NKG2A- Qa-1^b^ axis in ocular immune regulation and glaucoma progression, we generated D2 mice that are sufficient in CD94 (D2 *Klrd1*^*+/+*^). The wild-type CD94 *(Klrd1)* allele was backcrossed into present day D2 mice from the DBA/2 J-*Dtnbp1*^*sdy*^/J strain, which has the ancestral wild-type *Klrd1* allele that was lost from modern D2 mice (see Methods). D2 *Klrd*^*+/-*^ mice were intercrossed to produce *Klrd1*^*+/+*^, *Klrd1*^*+/-*^, and *Klrd1*^*-/-*^ littermates. We assessed expression of CD94 by labeling peripheral blood mononuclear cells with anti-CD94 and anti-NKG2 antibodies. Flow cytometric analysis demonstrates coexpression of CD94 and NKG2 in a subset of lymphocytes from D2 *Klrd1*^*+/+*^ mice but not D2 *Klrd1*^*-/-*^ mice (Figure 3).

**Figure 4:**
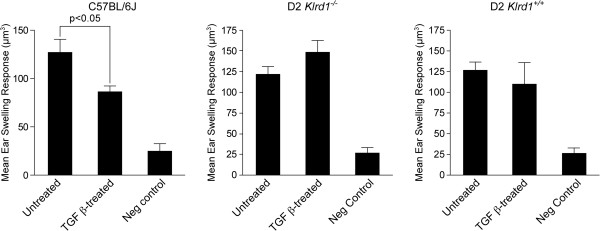
**Tolerogenic capacities of APCs from D2 *Klrd1***^***+/+***^**mice.** APCs isolated from mice of the indicated genotypes were injected into B6D2F1/J recipients. The APCs were first cultured either in absence (Untreated) or presence of TGFβ2 and pulsed with OVA. The ability of the APCs to induce immune deviation was determined by measuring the ear swelling response of F1 recipients to an OVA challenge (F1s had been previously inoculated with OVA). Functional immune deviation will result in suppression of a DTH response to the OVA challenge, resulting in no or minimum ear swelling. In contrast and if APCs are unable to manifest immune deviation, OVA challenge will induce ear swelling. Induction of immune deviation is detected as the suppression of ear swelling response in the TGFβ2-treated groups as compared to the untreated group. Ear swelling responses are presented as mean ± SEM. Negative controls (Neg control) were naïve and only received the intrapinnae challenge injection of OVA.

First, we determine if CD94 deficiency related changes in D2 APCs reflect a qualitative defect in their ability to induce ACAID. Non-ocular APCs, such as F4/80+ macrophages derived from thioglycollate-elicited peritoneal exudate cells, can be converted into APCs that induce immune deviation similar to that induced by ocular APCs. This is accomplished by exposing non-ocular APCs to TGFβ2 in culture. The resulting TGFβ2-exposed peripheral APCs resemble ocular APCs in their functional phenotype [22]. We compared the ability of TGFβ2 treated APCs derived from D2 mice that are either sufficient or deficient in CD94, to induce immune deviation by injecting them into recipient mice and thereafter assessing for inhibition of a DTH response. All experiments utilized genetically identical F1 mice from a cross between C57BL/6J and DBA/2J (B6D2F1) as recipients. It is important to note that the use of B6D2F1 recipient mice ensured expression of wild-type CD94 (*Klrd1*^*+/-*^*)* by host tissues. Thus, any observed differences are a direct consequence of an affect of CD94 genotype on ability of APCs to support ACAID. TGFβ2-treated APCs from control B6 mice successfully induced immune deviation that led to inhibition of DTH (Figure 4A). In contrast, APCs derived from either D2 *Klrd1*^*-/-*^ or D2 *Klrd1*^*+/+*^ mice both failed to inhibit DTH (Figure 4). Thus, irrespective of CD94 genotype, APCs derived from DBA/2J mice failed to induce ACAID. These results suggest that another inherent defect(s) in APCs (independent of CD94 or possibly acting with CD94) contributes to loss of ACAID and therefore immune privilege in DBA/2J mice.

### CD94 sufficiency does not impact the progression to high IOP and glaucoma

**Figure 5:**
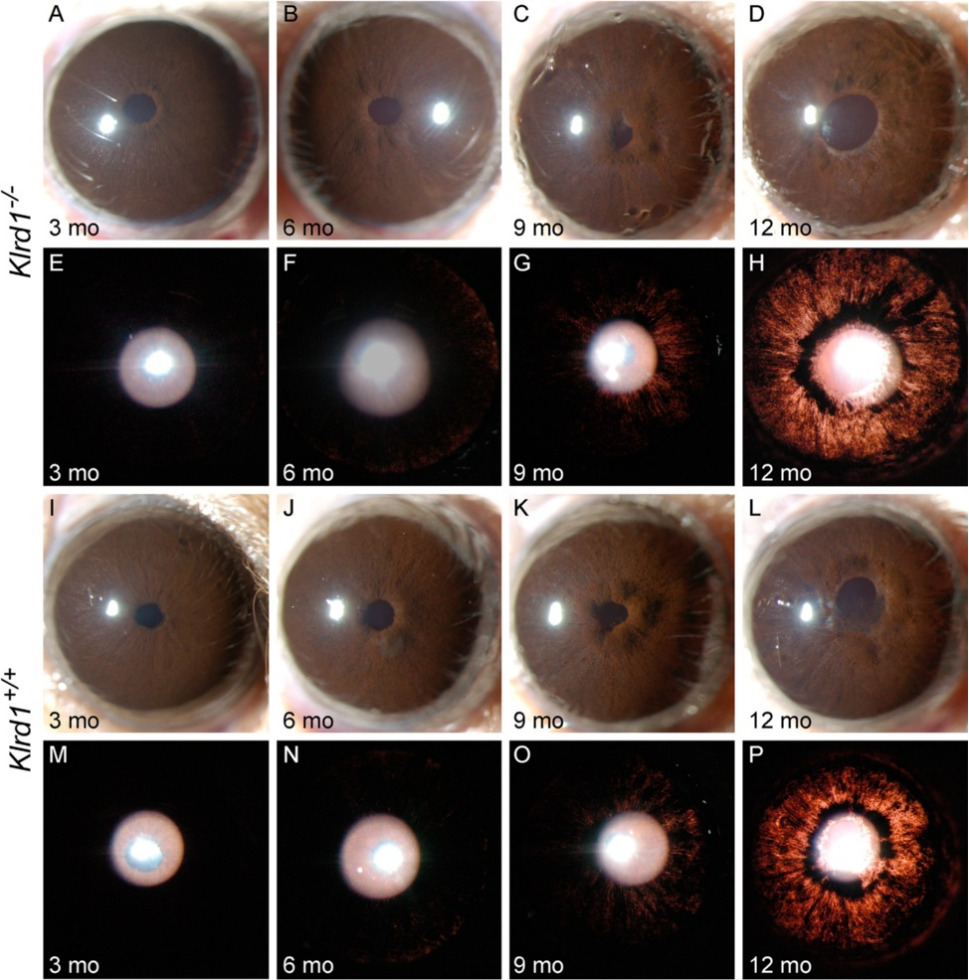
**CD94 status does not affect the iris disease in D2 mice.** Representative images for mice of each genotype are shown, with broad beam illumination on top of transillumination images. Comparative analysis of CD94 sufficient and deficient mice (*Klrd1*^*+/+*^and *Klrd1*^*-/-*^) indicates that the onset and progression of iris disease in D2 *Klrd1*^*-/-*^**(A-H)** is similar to that in D2 *Klrd1*^*+/+*^**(I-P)**.

**Figure 6:**
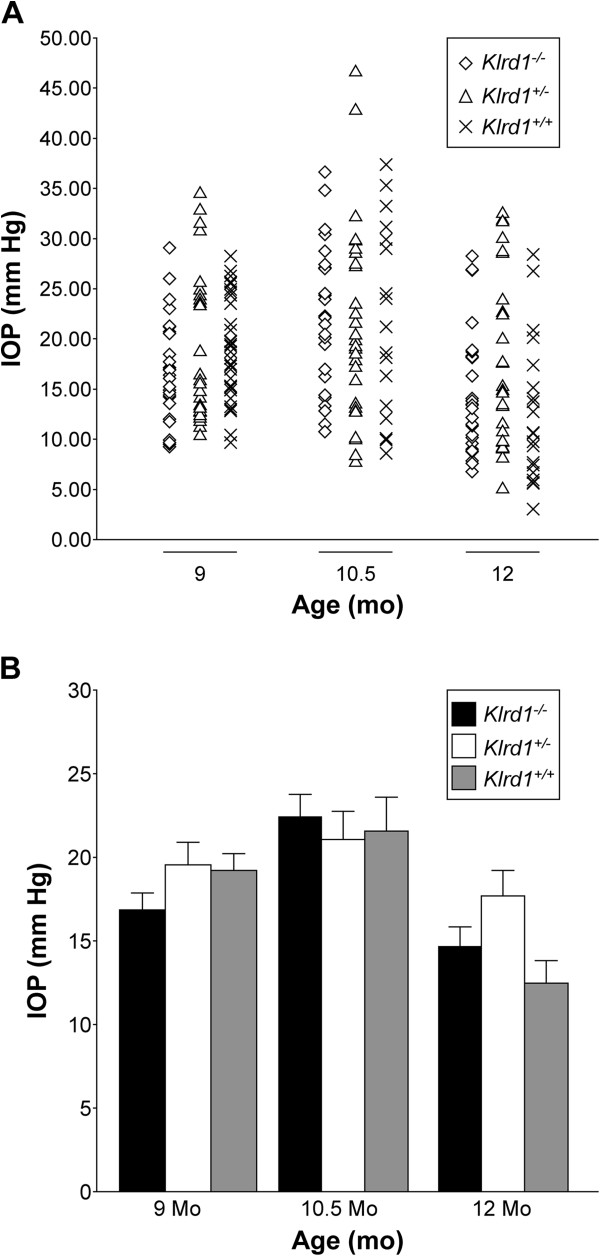
**CD94 sufficiency does not alter IOP in D2 mice.** In **(A)** the data is represented as a scatter plot, and in **(B)** as bar graphs of the mean IOP ± SEM (>30 mice). All groups contained at least 30 mice and included both males and females in similar proportions. Comparative analyses between the three genotypes showed that there were no significant differences in IOP (P > 0.05 for ages at 9 and 10 mo). At 12 mo time-point, only D2 *Klrd1*^*+/-*^ exhibited a slightly higher mean IOP (P = 0.046). Since IOP values between CD94 sufficient (D2 *Klrd1*^*+/+*^) and deficient (D2 *Klrd1*^*+/+*^) mice are comparable, this effect is unlikely to be dependent on genotype. A significant proportion of D2 mice typically show a drop in IOP at around 12 mo of age. The slight increase in mean IOP exhibited by D2 *Klrd1*^*+/-*^ mice is likely due to a high variability in IOP values as a consequence of their crashing in some mice. Age related ciliary-body atrophy is though to contribute to this IOP drop at around 12 mo in D2 mice.

We tested the effect of CD94 sufficiency on the disease progression to glaucoma. D2 mice of all three genotypes (*Klrd1*^*+/+*^, *Klrd1*^*+/-*^, and *Klrd1*^*-/-*^) were aged and assessed. CD94 sufficiency has no affect on the age of onset, progression, or severity of pigment dispersing iris disease (Figure 5). We next assessed whether the CD94 sufficiency alters IOP in D2 mice. Mice of all three genotypes were aged and their IOP determined. The IOP elevation in DBA/2J mice peaks from 9 to 12 mo of age, with the IOP distribution clearly shifting upward between 8 and 9 mo. We monitored IOP at key ages (9, 10.5, and 12 mo). Our data demonstrate that IOPs of all three genotypes were not significantly different at the recorded ages (Figure 6 A, B). Our results indicate that CD94 sufficiency did not alter the high IOP phenotype exhibited by D2 mice. Thus, the CD94 deficiency of modern DBA/2J mice does not influence either the iris disease or IOP elevation.

**Figure 7:**
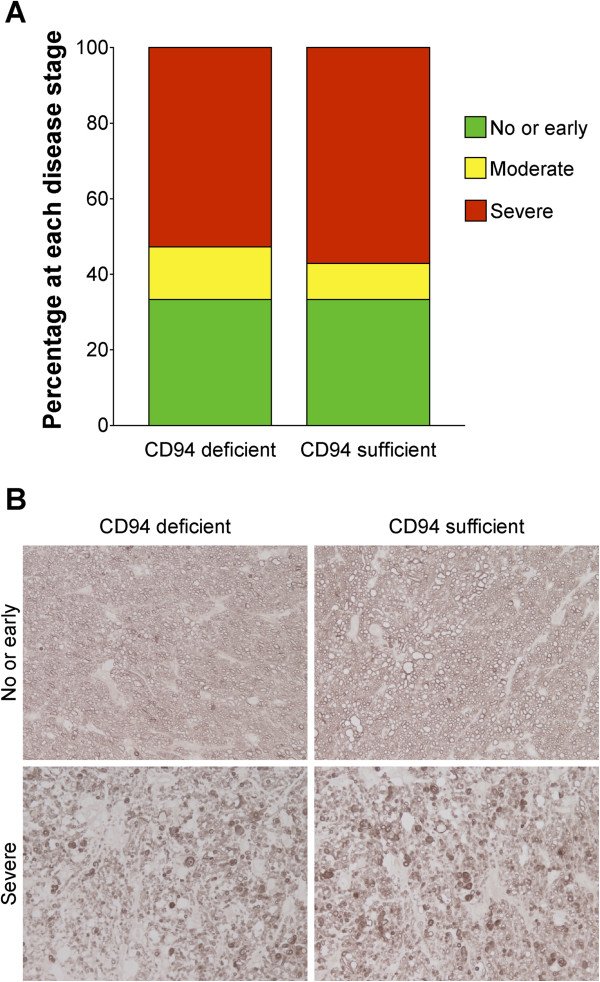
**Degree of optic nerve degeneration is comparable between CD94 sufficient and deficient mice.** To assess the effects of CD94 sufficiency on optic nerve degeneration, we analyzed PPD-stained optic nerve cross sections. For this analysis, CD94 sufficient mice had either a D2 *Klrd1*^*+/+*^*or* D2 *Klrd1*^*+/-*^ genotype, while CD94 deficient mice were *Klrd1*^*-/-*^*.***A)** Distributions of optic nerve damage at 12 mo. **B)** Representative images from nerves with no glaucoma have a clear axoplasm and darkly stained myelin sheath. Nerves with severe damage have extensive axon loss and glial scarring. CD94 status did not alter glaucomatous optic nerve damage. For each group > 30 eyes were evaluated.

Autoimmune mechanisms have been suggested to contribute to retinal ganglion cell death in glaucoma. Immune mediated damage to the optic nerve has been hypothesized to result from responses elicited against a sensitizing antigen, which in turn injures retinal ganglion cells. Although not clearly defined, there is evidence to support involvement of both innate and adaptive immune responses in glaucomatous neuropathy [35]. In D2 mice, severe glaucomatous damage is detectable in about 70% of 12-month-old mice, at this stage the degree of glaucomatous damage almost reaches saturation, with little damage occurring at older ages [11]. The frequency and nature of glaucomatous nerve damage was similar between D2 mice deficient and sufficient in CD94 (Figure 7A, B). Thus, CD94 deficiency does not alter the ultimate incidence and severity of optic nerve degeneration.

## Discussion

D2 mice develop a form of pigmentary glaucoma involving a pigment dispersing iris disease, increased IOP, and degeneration of retinal ganglion cells. Our earlier studies suggest that melanosomal defects along with ocular immune abnormalities participate in the progression to D2 glaucoma. Here, we demonstrate that innate immune processes mediated through NK cells are not important for the progression of the iris disease. In addition, we rule out natural deficiency of CD94 as a pathogenic factor necessary for progression of distinct disease stages that underlie D2 glaucoma.

### Innate immune components other that NK cells are involved in propagation of the iris disease

We have previously reported that reconstitution of irradiated D2 mice with B6D2F1 bone marrow rescues the prominent pigment dispersion phenotype that is caused by mutations in *Tyrp1* and *Gpnmb*[21]. This rescue is mediated through suppression of ocular immune abnormalities detectable early in the disease. Subsequently, we have identified the *Gpnmb* gene as an important factor that mediates this bone marrow derived rescue of this phenotype [22]. Based on this, we had speculated that a bone marrow derived lineage that would normally express *Gpnmb* reacts abnormally to the iris debris in D2 mice. As a consequence of GPNMB deficiency, the bone marrow derived cells may either fail to inhibit an ocular inflammation or may directly inflict an inflammatory attack on the iris, causing severe pigment dispersion and iris atrophy. We have previously ruled out components of adaptive immunity (T and B cells) as necessary in the propagation of *Tyrp1* and *Gpnmb* induced iris disease [22]. Therefore, these phenotypes are likely dependent on cell types regulating innate immune responses. Here, we demonstrate that deficiency of NK cells - an important component of innate immunity - had no affect on the propagation of the iris disease.

Other mediators of innate immune responses, including macrophages and dendritic cells, express GPNMB [17]. Importantly, GPNMB acts as a negative regulator of macrophage mediated inflammatory responses [17]. Macrophages are normally resident in the anterior chamber of the eye and large numbers of pigment-containing macrophages are visible in the anterior chamber of aged D2 eyes. As a consequence of the *Gpnmb* mutation, D2 macrophages may acquire a proinflammatory function and inflict damage to the iris. Future studies will focus on understanding the role of macrophages in the progression of the D2 iris disease.

### Deficiency of CD94 does not contribute to progression to D2 glaucoma

Our earlier study has suggested that the inability of D2 mice to support ACAID, a form of ocular immune tolerance, may contribute to both the immune dysfunction and glaucoma progression that occurs in D2 eyes [21]. A recent report has shown a causal relationship between CD94/NKG2A receptor and its ability to support ACAID [26]. Ligation of CD94/NKG2A expressed on a subset of CD8 Tregs is critical for induction and immunosupression in ACAID [26,36]. This implies that failure of D2 mice to support ACAID is partly due to deficiency of CD94 [26].

Ocular APCs are thought to be the primary initiator of the ACAID response. They act by capturing antigen in the eye and migrating to the spleen, where they initiate a poorly understood, complex series of cellular processes involving interplay of multiple immune cell types including B cells and CD8 Tregs [34,37]. We have previously reported that APCs isolated from D2 mice fail to support ACAID. This is in agreement with another study that suggest defects in multiple immune cell-types contribute to a faulty D2 ACAID response [26]. Here, we demonstrate that the APCs derived from CD94 sufficient DBA/2J mice continue to exhibit an inherent defect and fail to support ACAID. Insight into factors controlling the multiplicity of defects in different immune cell types will expand our understanding of the molecular mechanism controlling ACAID and ocular immune privilege.

Similarly, a correction of CD94 deficiency alone did not alter the iris disease, high IOP or degree of optic nerve degeneration exhibited by D2 mice. The etiology of IOP elevation and glaucoma in D2 mice is clearly complex, involving multiple factors. Although mutations in *Gpnmb* and *Tyrp1* are sufficient to cause iris disease, our previous work indicates that additional D2 specific alleles at other loci are needed for progression to high IOP and glaucoma [31]. Here, we have ruled out CD94 deficiency as a factor necessary for iris disease or progression to high IOP and glaucoma in D2 mice. Efforts are underway to identify the additional genes that are necessary for high IOP and to characterize their role in the progression from pigment dispersion to IOP elevation and glaucoma.

## Conclusions

Here, we have demonstrated that NK cells are not necessary for the progression of *Tyrp1*^*b*^*Gpnmb*^*R150X*^ driven iris disease. In addition, we rule out CD94 deficiency as an important factor contributing to pathogenesis of D2 glaucoma. Future studies will attempt to identify immune-related susceptibility factors that control D2 glaucoma. Characterizing the genes and mechanisms that underlie this progression will identify new pathways that can be evaluated in human pigmentary glaucoma, and may provide valuable therapeutic targets.

## Methods

### Animal husbandry

Mice were obtained from The Jackson Laboratory (Bar Harbor, ME, USA). All animals were treated according to the guidelines of the Association for Research in Vision and Ophthalmology for use of animals in research. All experimental protocols were approved by the Animal Care and Use Committee of The Jackson Laboratory. The DBA/2J (D2) mice were maintained on an NIH 31 diet with a fat content of 6%. To avoid obesity, mice with a C57BL/6J (B6) background were maintained on essentially the same NIH 31 diet but with a 4% fat content. Our studies have shown that D2 IOP and other glaucoma phenotypes do not differ with this small dietary difference (unpublished data). For all mice, the diet was provided *ad libitum*, and water was acidified to pH 2.8–3.2. Mice were housed in cages containing white-pine bedding and covered with polyester filters. The environment was kept at 21°C with a 14-hour light: 10-hour dark cycle.

### Generation of strains

Mice with a perforin null allele (B6-*Prf1*^*tm1Sdz*^/J) (stock # 2407) were obtained from The Jackson Laboratory and crossed to a congenic B6.D2-*Gpnmb*^*R150X*^*Tyrp1*^*b*^ strain [31] to produce mutants that were homozygous for all three mutations (B6.Cg-*Prf1*^tm1Sdz^*Tyrp1*^*b*^*Gpnmb*^*R150X*^/Sj), referred to as the perforin mutant. In addition, mice carrying IL2 receptor gamma chain null mutation (B6-*Il2rg*^*tm1Wjl*^*/J*) were obtained from the Jackson Laboratory and crossed to the B6.D2-*Gpnmb*^*R150X*^*Tyrp1*^*b*^ strain to generate B6.Cg- *Il2rg*^*tm1Wjl*^*Tyrp1*^*b*^*Gpnmb*^*R150X*^/Sj mice, referred to as the *Il2rg* mutant. The *Klrd1* (CD94) mutation in D2 mice was determined to have occurred between 1984 and 1989. The *sdy* mutation alters coat color and occurred in D2 mice in 1983. The DBA/2J-*Dtnbp1*^*sdy*^/J strain (D2-sdy) was separated from the main D2 colony. Genotyping a D2-sdy colony for *Klrd1* revealed that these mice had an original wild-type allele. To develop the *Klrd1*^*+/+*^ strain, D2-sdy was crossed to modern D2 mice for three generations. Congenic D2 *Klrd1*^*+/-*^ mice were intercrossed to produce *Klrd1*^*+/+*^, *Klrd1*^*+/-*^, and *Klrd1*^*-/-*^ littermates. All three genotypes were housed together and analyzed simultaneously.

### Flow cytometry

Peripheral blood mononuclear cells were stained with monoclonal antibodies against CD94 and NKG2A/B/C (The Jackson Laboratory Flow Cytometry Service). Labeling was assessed by multicolor flow cytometry (FACScan™ or FACScalibur®; BD Biosciences) and analyzed using the CellQuest 3.3 data reduction system (BD Biosciences).

### Assay for Anterior Chamber Associated Immune Deviation (ACAID)

Peritoneal Exudate Cells (PECs) obtained 3 days after i.p. injection of mice with 2 ml of 3% thioglycollate solution (Sigma Chemical Co., St. Louis, MO) were cultured overnight in serum free medium with ovalbumin (OVA) antigen (7 mg/ml) in the presence or absence of TGFβ2 (5 ng/ml). Cells were then harvested and washed three times with cultured medium to remove excess TGFβ2 and non-adherent cells. Adherent cells that typically contain >90% F4/80+ and >99% CD11b + cells [38] served as antigen presenting cells (APCs). These cells (2–5 x 10^3^/mouse) were infused i.v. in recipients immunized s.c. 7 days earlier with 50 μg OVA in Complete Freund’s Adjuvant. A week later these mice received intradermal inoculation of OVA (200 μg/20 μl) in their right ear pinnae while left ears served as untreated controls. Thickness of both ears was measured immediately before and at 24 hr interval after the OVA injection using a micrometer (Mitutoyo, MTI Corp., Paramus, NJ). The measurements were performed in triplicates. Delayed type hypersensitivity (DTH) was measured as a change in ear swelling ([24-hr measurement – 0-hr measurement in the experimental right ear] – [24-hr measurement – 0-hr measurement in the control left ear]). Suppression of DTH in the experimental group was determined by comparison with the DTH response detected in a group receiving untreated APCs. A two-tailed Student’s t-test was used to assess significance.

### Clinical examination

Eyes were examined with a slit-lamp biomicroscope and photographed with a 40× objective lens. Assessment of iris disease was determined by indices of iris atrophy and dispersed pigment following previously described criteria [22]. Transillumination defects were also measured (transillumination is an assay of iris disease whereby reflected light passing through depigmented areas of iris tissue are visualized as reddish light). Examination of at least 20 mice of each genotype at 3, 6 and 9 months of age, and at the time of harvest at 12 months, was performed. Additionally, smaller groups of mice (10–20 of each genotype) were analyzed every month between 3 and 12 month of age.

### IOP measurement

IOP was measured using the microneedle method as previously described in detail [39]. All cohorts included male and female mice. B6 mice were interspersed with experimental mice during all experiments as a methodological control to ensure proper equipment calibration and performance. Analysis of Variance (ANOVA) was used to test significance.

### Optic nerve assessment

The majority of optic nerves were collected from mice within 48 hours of IOP measurement. Samples from both males and females, as well as left and right nerves were used for analysis. Optic nerves were dissected, processed, embedded in plastic, sectioned and stained with paraphenylenediamine (PPD) as previously described. PPD stains myelin sheaths of all axons and stains the axoplasm of sick or dying axons darkly. Stained sections were compared using a previously described qualitative grading [40].

## Competing interests

The authors declare that they have no competing interests.

## Authors’ contributions

KSN, SM and SWMJ conceived experiments, analysed the data and prepared the manuscript. SWMJ oversaw all aspects of the study. KSN and JB bred the mice and performed clinical analysis of mice. KSN performed flowcytometric analysis. SM performed ACAID analysis. RS performed optic nerve assessment. All authors read and approved the final manuscript.
